# Performance of Temporal and Spatial Independent Component Analysis in Identifying and Removing Low-Frequency Physiological and Motion Effects in Resting-State fMRI

**DOI:** 10.3389/fnins.2022.867243

**Published:** 2022-06-10

**Authors:** Ali M. Golestani, J. Jean Chen

**Affiliations:** ^1^Department of Psychology, Toronto Neuroimaging Facility, University of Toronto, Toronto, ON, Canada; ^2^Rotman Research Institute at Baycrest, Toronto, ON, Canada; ^3^Department of Medical Biophysics, University of Toronto, Toronto, ON, Canada; ^4^Institute of Biomedical Engineering, University of Toronto, Toronto, ON, Canada

**Keywords:** fMRI, resting-state, temporal ICA, spatial ICA, head motion, physiological noise

## Abstract

Effective separation of signal from noise (including physiological processes and head motion) is one of the chief challenges for improving the sensitivity and specificity of resting-state fMRI (rs-fMRI) measurements and has a profound impact when these noise sources vary between populations. Independent component analysis (ICA) is an approach for addressing these challenges. Conventionally, due to the lower amount of temporal than spatial information in rs-fMRI data, spatial ICA (sICA) is the method of choice. However, with recent developments in accelerated fMRI acquisitions, the temporal information is becoming enriched to the point that the temporal ICA (tICA) has become more feasible. This is particularly relevant as physiological processes and motion exhibit very different spatial and temporal characteristics when it comes to rs-fMRI applications, leading us to conduct a comparison of the performance of sICA and tICA in addressing these types of noise. In this study, we embrace the novel practice of using theory (simulations) to guide our interpretation of empirical data. We find empirically that sICA can identify more noise-related signal components than tICA. However, on the merit of functional-connectivity results, we find that while sICA is more adept at reducing whole-brain motion effects, tICA performs better in dealing with physiological effects. These interpretations are corroborated by our simulation results. The overall message of this study is that if ICA denoising is to be used for rs-fMRI, there is merit in considering a hybrid approach in which physiological and motion-related noise are each corrected for using their respective best-suited ICA approach.

## Introduction

Functional MRI (fMRI) is a powerful tool to non-invasively investigate brain function and organization. However, several confounding noise sources typically affect the sensitivity and specificity of associated results, chiefly coming from physiological processes and bulk head motion. These nuisance effects typically need to be removed to reduce false positive and false negative results, particularly in resting-state fMRI (rs-fMRI). Desirable clean-up methods should selectively remove noise while preserving signals of interest generated by presumed neural activity (Murphy et al., 2013; Caballero-Gaudes and Reynolds, 2017). Identifying and removing physiological noise such as those induced by temporal variability in respiratory volume and heart rate (Birn et al., 2006; Chang et al., 2009; Golestani et al., 2015), as well as by head motion (Power et al., 2012, 2015; Yan et al., 2013; Maknojia et al., 2019) are particularly challenging. While the higher-frequency respiration and cardiac cycles have been better characterized and found easier to correct (Glover et al., 2000), the low-frequency physiological effects have characteristics that vary among different subjects and populations. Moreover, subtle head motion, which is likely connected to such physiological effects in no small part, is notoriously hard to identify and remove (Van Dijk et al., 2012).

Several studies have used independent component analysis (ICA) to remove the effects of physiological signals from fMRI (Salimi-Khorshidi et al., 2014; Pruim et al., 2015). ICA is a data-driven method that can be used to identify physiological components of the fMRI signal without *a priori* knowledge about their dynamics or additional equipment to record the physiological signals. Since fMRI data typically has more voxels than time-points, so far it has been more feasible to perform spatial ICA (sICA) on fMRI data (Smith et al., 2012). Indeed most of the available ICA-based data-cleaning tools are based on sICA (Griffanti et al., 2014; Salimi-Khorshidi et al., 2014; Pruim et al., 2015). To generate N components from an ICA, typically kN^2^ data points are required, where k is the number of data points per component, and k > 2. For e.g., if we seek to generate 20 components in a tICA, then we may need 800 time points, which is unavailable in most rs-fMRI scans. Conversely, as fMRI has larger spatial dimension (voxels) than temporal dimensions (time-points), sICA is conventionally more common in fMRI and generates more reproducible results than tICA. Therefore, sICA generates more accurate and reproducible results. However, recent developments in multiband data acquisition enable acquiring fMRI with higher temporal resolutions, which makes temporal ICA (tICA) more feasible (Calhoun et al., 2001; Stone et al., 2002; Chen et al., 2006; Penney and Koles, 2006; Wang et al., 2006; Lukic et al., 2007; van de Ven et al., 2009; Alkan et al., 2011; Gao et al., 2011; Boubela et al., 2013; Miller et al., 2014; Hald et al., 2017; Shi and Zeng, 2018; Amemiya et al., 2019; Baker et al., 2019). tICA has begun to be used in noise identification in rs-fMRI (Beall and Lowe, 2007; Glasser et al., 2018, 2019; Power, 2019). Regardless, sICA is still the method of choice for rs-fMRI noise removal.

Previous studies indicate inherent differences in denoising performance by tICA and sICA. Although sICA can successfully identify spatially localized fluctuations, it likely fails to separate spatially global components (Glasser et al., 2018) with spatially overlapping sources (Smith et al., 2012; Boubela et al., 2013). Specifically Calhoun et al., (Calhoun et al., 2001) have shown that sICA and tICA fail in separating underlying components if the components are spatially and temporally inter-dependent, respectively. The deficiency of sICA in fMRI clean-up has been demonstrated (Burgess et al., 2016; Siegel et al., 2017), and tICA has shown promising results in identifying physiological noise in the fMRI data. Boubela et al. (2013) were able to identify physiological signals such as cardiac pulsation using tICA. Moreover, Glasser et al. (2018) used tICA as a replacement for the controversial global signal regression and showed tICA can identify and remove global fluctuations in the fMRI data (which presumably is due to physiological nuisance) while preserving neural signals. However, in both studies, the data from multiple subjects were concatenated and a group-wise tICA on the concatenated data was performed. Therefore, it is not clear how tICA performs in terms of a single-subject ICA. This poses a major limitation, as for the fMRI to be clinically useful, it should ideally be an individualized metric. Moreover, the study by Glasser et al. (2018) mainly focused on identifying a component associated with the global signal and did not investigate how tICA performed in identifying and removing specific physiological noise effects.

Intuitively, tICA can better distinguish between temporally independent but spatially correlated components (Calhoun et al., 2001; Boubela et al., 2013; Glasser et al., 2018) compared to sICA. These include effects of low-frequency physiological fluctuations, which could encompass the well-known effects of respiratory variability (RVT), heart-rate variability (HRV), and end-tidal carbon dioxide (PETCO_2_), potentially overlapping with each other spatially and temporally (Tong et al., 2019; Bright et al., 2020). Some of these physiological signals are also temporally related to one another (Chang and Glover, 2009; Glasser et al., 2018; Power et al., 2019b). On the other hand, some noise sources are spatially less restricted and overlap with the spatial pattern of other noises as well as with several resting-state networks (Griffanti et al., 2014; Salimi-Khorshidi et al., 2014; Golestani et al., 2015). It is unclear if ICA (either temporal or spatial) can identify and separate all of these sources of noise.

In this study, we compare the performance of sICA and tICA in identifying and removing physiological noises on a single-session (non-concatenated) basis. The first objective of this study is to investigate if sICA and tICA can identify different noise components in different ICs. Since different noises have spatial or temporal dependencies, we hypothesize that tICA and sICA perform differently in identifying different physiological components of the resting-state fMRI (rs-fMRI) signal. The second objective of this study is to compare tICA and sICA performance in removing noise from the fMRI data while preserving the information about brain function. It is not immediately obvious which would excel in the preservation of neuronal information.

## Theory

### ICA

Independent component analysis is a method for decomposing multivariate linearly combined signals into its components, assuming the components are statistically independent and non-Gaussian. Assuming we observe *m* signals *X = (x_1_, …,x_*m*_)*^T^**, which are a linear mixture of *n* hidden components *S = (s_1_, …, s_*n*_)*^T^**. The mixture can be written as a matrix multiplication as follow:


(1) X=M×S

where *M* is an *m × n* mixing matrix. Assuming the components in *S* are statistically independent, ICA tries to estimate a separating matrix *W* so that


(2) $$W=M^{-1}$$

Using *W*, we can estimate the original components:


(3) $$\hat{S}=W\times X$$

### Spatial ICA

To implement spatial ICA (Beckmann et al., 2005) on fMRI data, the data is first reordered into a 2-dimensional matrix of time x space. Assuming we have *n* voxels with *t* time samples, the fMRI data can be modeled as:


(4) $$Data_{t\times n}^{s}=M_{t\times c}^{s}\times S_{c\times n}^{s},$$

where *S^s^* is a *c × n* matrix of *c* spatially independent components and *M^s^* is a mixing matrix that consists of temporal signatures of the spatial components. Note that in this case we assume that the data consists of *c* spatially independent components. Using sICA we estimate the separating matrix as


(5) $$W^{s}={\left(M_{t\times c}^{s}\right)}^{-1}$$

Using the separating matrix, we estimate the spatial components ${\hat{S}}_{c\times n}^{s}$:


(6) $${\hat{S}}_{c\times n}^{s}=W_{c\times t}^{s}\times Data_{t\times n}^{s}$$

Time courses of the components can be estimated by calculating the pseudo-inverse of the separating matrix *W^s^*.

### Temporal Independent Component Analysis

To perform temporal ICA (Smith et al., 2012; Boubela et al., 2013; Salimi-Khorshidi et al., 2014; Glasser et al., 2018), the fMRI data is transposed into a *n × t* matrix. Therefore, the data can be modeled as:


(7) $$Data_{n\times t}^{t}=\;M_{n\times c}^{t}\times S_{c\times t}^{t}$$

The three matrices of *Data^t^*, *M^t^* and *S^t^* are the transpose of *Data^s^*, *M^s^* and *S^s^* for the spatial ICA case. We assume that the components time series in *S^t^* are independent and we try to estimate the separating matrix


(8) $$W^{t}={\left(M_{n\times c}^{t}\right)}^{-1}$$

Then we can estimate the components time courses ${\hat{S}}_{c\times t}^{t}$:


(9) $${\hat{S}}_{c\times t}^{t}=W_{c\times n}^{t}\times Data_{n\times t}^{t}$$

The spatial maps can be estimated by calculating the pseudo-inverse of the separating matrix *W^t^*.

## Materials and Methods

### Simulations

**FIGURE 1:**
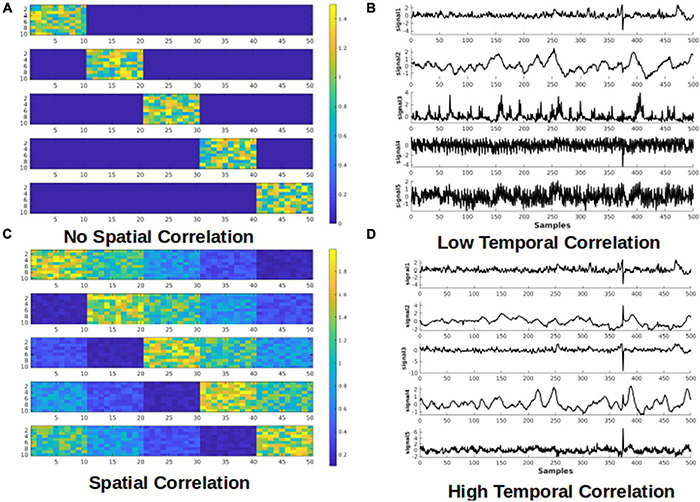
Simulated test cases. Examples of spatial **(A,C)** and temporal **(B,D)** components used for the simulation. Examples of the spatial maps of the five components are presented on the left. Each component has dimensions of 10 × 50. Examples of temporal signals of the five components are presented on the right. Each component has 500 time samples (time points). Panels **(A,B)** show the cases that the components have low correlation, whereas the panels **(C,D)** represent the cases where the components are highly correlated.

To guide the formation of our hypothesis regarding the effectiveness of sICA and tICA in identifying noise ICs, simulations were first performed, in which the fMRI data is simulated as a mixture of known components (ground truth). The same methodology has been used previously to simulate task-based fMRI (Calhoun et al., 2001). Each of the simulated datasets consists of five components of interest with known spatial and temporal patterns, as shown in Figure 1. Signals of the five components of interest are taken from an *in vivo* fMRI dataset used in this work (acquisition details to follow). The *in vivo* fMRI dataset is decomposed into 50 ICs using the spatial ICA algorithm implemented in MELODIC, and for computational simplicity, only the initial 500 time points of each component are used. The spatial map of the simulated data consists of 500 voxels in a 2-dimensional matrix with 10 × 50 voxels (which can be conveniently divided into 5 sub-regions, each with a dimensionality of 10 × 10). In addition, one component with random spatial and temporal patterns is added to represent noise. Therefore, the mixing matrix *M* has dimensions of 500 × 6 (500 time samples and 6 components, i.e., 5 temporal components of interest and 1 noise component), and the components matrix S is a 6 × 500 matrix (500 voxels and 6 spatial components, i.e., 5 spatial components of interest and 1 random “noise” spatial patterns). Thus, the final resultant dataset has a dimensionality of 500 × 500. These components were used to produce four datasets to represent four different scenarios (Figure 1).

1. Spatially and temporally uncorrelated components; emulates random noise such as from thermal sources; We predict that tICA and sICA are agnostic in identifying these components.
2. Spatially correlated components with low temporal correlation; emulates spatially correlated but temporally asynchronous processes, such as visual activity and respiration in the occipital cortex (Birn et al., 2006; Golestani et al., 2015). We hypothesize tICA performs better in identifying these components.
3. Temporally correlated components with minimal spatial correlation; emulates spatially localized but temporally synchronous events, such as different nodes of a brain network or head motion is a specific direction; sICA assumes to be more successful in identifying such components.
4. Spatially and temporally correlated components: this is the most interesting and challenging case, in which we presume neuronal and vascular signal sources coincide both temporally and spatially (Bright et al., 2020). The performance of tICA and sICA in identifying these components is unclear.

Regarding the component signals, for Scenarios 1 and 2, five signals are randomly selected from a set of 10 signals with the lowest mutual temporal correlation (the average absolute correlation among the 10 components = 0.0325), which reflects high independence among the temporal components. To model high temporal dependence for scenarios 3 and 4, five signals are randomly selected from a pool of 10 highly mutually correlated signals (average absolute correlation among the 10 signals = 0.2701). Regarding the spatial components, for scenarios 1 and 3 (low spatial correlation), each signal is added to only one of the five sub-regions, whereas for scenarios 2 and 4 (high spatial correlation), each signal is added to all five sub-regions with different weightings. Examples of the components’ time-courses with low and high temporal correlation are shown in Figures 1B,D. To the best of our knowledge, this is a novel framework for determining the effectiveness of ICA-based methods for separating rs-fMRI-relevant signals contributions.

Datasets are generated by multiplying the spatial (*M*) and temporal (*S*) matrices. 100 datasets are generated for each scenario by varying the random noise, voxel values of the spatial patterns, and randomly selecting 5 out of 10 time-courses. Each dataset is then decomposed into its components using both sICA and tICA. The performance of the ICA algorithms is measured by comparing the spatial and temporal correlation between the 5 original and the 5 ICA-identified components. In each simulation, the correlation between the five identified components and the five original signals is calculated and then the five correlation values are averaged to estimate an overall correlation between the original signals and the identified component. We realize that the assumption of temporally and spatially randomness for the noise component is an over-simplification in fMRI, but the goal of these simulations is to demonstrate the differential effects of tICA and sICA on temporally or spatially correlated signal components. The hypothesis is that while tICA should be better at separating temporally dissociated but spatially correlated signal components, the converse should be true for sICA.

### Data Acquisitions

Nineteen healthy subjects (age = 26.5 ± 6.5 years) were scanned using a Siemens TIM Trio 3T MRI scanner with a 32-channel head coil. rs-fMRI scans were collected using simultaneous multi-slice GE-EPI BOLD (TR/TE = 380/30 ms, flip angle = 40°, 20.5-mm slices, 64 × 64 matrix, 4 mm × 4 mm × 5 mm voxels, multiband factor = 3, 1,950 volumes). During each scan, end-tidal CO_2_ pressure (PETCO_2_) fluctuations were passively monitored using a RespirAct™ system (Thornhill Research, Toronto, Canada). In addition, cardiac pulsation was recorded using the Siemens scanner pulse oximeter (sampling rate = 50 Hz), whereas the respiratory signal was recorded using a pressure-sensitive belt connected to the Biopac™ (Biopac Systems Inc., CA, United States) at a sampling rate of 200 Hz. A T1-weighted anatomical image was also collected (MPRAGE, TR = 2,400 ms, TE = 2.43 ms, FOV = 256 mm, voxel size = 1 mm × 1 mm × 1 mm).

### Preprocessing and ICA

The rs-fMRI processing pipeline includes: motion correction, spatial smoothing (Gaussian kernel with 5 mm FWHM), and high-pass filtering (>0.01 Hz). We chose to estimate 30 ICs, as this is a typical number of components used in the literature that provides a good trade-off between providing a good representative of the fMRI data structure and making the analysis and interpretation more manageable (Wang and Li, 2015; Vergun et al., 2016). For sICA, fast ICA (Hyvärinen, 1999) is used to divide fMRI data into 30 spatial components. For tICA, as is typical, the data dimension is first reduced to 100 components using sICA, and then tICA (Smith et al., 2012; Glasser et al., 2018) is performed on the 100 time-series to generate 30 temporal components. To assess the generalizability of our findings, we also obtained results when the signal was decomposed into 50 ICs (Supplementary Material).

### Markers of Noise: Physiological Variations and Motion

We address the signal contribution by different noise types, categorized as:

• Global physiological fluctuations, including PETCO_2_, RVT, and HRV, which have network structure and are spatially selective, but have temporal signatures that are distinct from those of neuronally driven BOLD signals. Heart-rate variation (HRV) is calculated as the average heart rate over a 4-s window (Chang et al., 2009). Respiratory-volume variability (RVT) is defined as the ratio of breathing depth to breathing period (Birn et al., 2006; Chang et al., 2009). PETCO_2_ is calculated by finding the peak PCO_2_ level in each breathing cycle and repeating over the entire tracing (Golestani et al., 2015). Subject-specific response functions for PETCO_2_, RVT, and HRV are obtained from the whole-brain global signal using the Gaussian-constrained maximum-likelihood deconvolution model (Falahpour et al., 2013; Golestani et al., 2015). In this study, to ensure fairness of comparisons, the physiological signals are convolved with the corresponding response function (i.e., PETCO_2_-conv, RVT-conv and HRV-conv).
• Global motion parameters, including framewise displacement (FD), the spatial root-mean-square of the time series (DVARS), the slow variations (SVAR). FD is calculated using FSL, as the sum of the absolute values of the derivatives of the six motion parameters. DVARS and SVAR are estimated using a MATLAB script (Jenkinson et al., 2002; Afyouni and Nichols, 2018). Specifically, DVARS is proportional to the sum of the squared framewise fMRI signal change and is weighted towards the fast portion of signal change. Conversely, SVAR is computed as the sum of the squared sum between consecutive fMRI frames and reflects the slow portion of signal change (Jenkinson et al., 2002; Afyouni and Nichols, 2018).
• Local motion parameters, including the six affine head motion parameters (three rotations and three translations). Bulk-motion time series, whether fast or slow, are expected to exhibit statistical properties that differ from non-motion signal substrates both temporally and spatially. The six affine motion parameters were generated using FSL’s MCFLIRT motion correction algorithm (Jenkinson et al., 2002).

### Evaluation Methods

**FIGURE 2:**
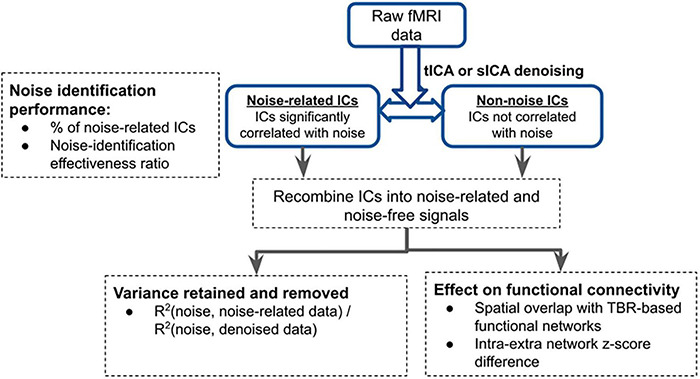
Overview of analysis pipeline for evaluating the performance of tICA and sICA. The performance of the two approaches are compared in three ways: (1) noise identification: the associated metrics are extracted from the results of the ICA step; (2) functional connectivity: these are extracted after combining noise-related and noise-free components to create noise and cleaned datasets; (3) variance explained: the amount of variance (of the original data) that is removed from the pre-ICA signal (as noise) and how much is retained (noise-free), as well as the variance accounted for by the noise markers.

Evaluation of sICA and tICA for separating signal and noise are evaluated using the following evaluation approach, using the noise (physiological variability and motion) markers described earlier. In all cases, signal contributions associated with each noise marker are obtained by combining all ICs that are significantly correlated with each noise marker. Conversely, the remaining ICs are combined to synthesize the non-noise related contribution for each noise type, respectively. Our methodology is detailed in Figure 2, and the evaluation rubrics are shown in Table 1.

**TABLE 1:** Rubric demonstrating how to interpret the performance of noise identification (A) and correction (B) based on the presented data.

Evaluation of noise detection performance		Evaluation of noise removal performance	
Noise-identification effectiveness ratio: R^2^ (noise-related ICs, noise markers) / R^2^ (non-noise ICs, noise markers)	Ideally high	Denoising-effectiveness ratio: Variance explained by noise markers in noise-related ICs / Variance explained by noise markers in denoised signal	Ideally high
		Spatial overlap with known functional networks (Dice_non_noise)	Ideally high
		Intra-extra network connectivity difference (ΔZ_non_noise)	Ideally high

*(A) A good noise identification consists of noise components with a strong correlation with the noise and a low correlation between the remaining components and the noise. On the contrary, a case of bad noise identification is when components are correlated with the noise time series and the correlation between the noise and noise-related components is comparable with the correlation between the noise and noise-free components. (B) Noise correction performance is evaluated by comparing the variance in the original data (before noise correction) and the variance in the cleaned and noise data explained by each noise marker. A good noise correction should reduce the R^2^ in the denoised data while having a high R^2^ in the noise data. Moreover, the Dice coefficient between rs-network templates and the components should stay the same or increase in the corrected data. Finally, ΔZ (the difference between the within-network and between-network Z-values) should not decrease after noise correction in the corrected data.*

### Noise Identification: Noise Content in Noise-Correlated ICs

To assess the extent of a given IC indeed being mostly noise, the correlation between each noise time series and the time course of each of the 30 temporal/spatial ICs is calculated. To assess the significance of the correlations, a null distribution is generated by calculating the correlation between a specific component and 5,000 permutations of the noise time course (to maintain the same power spectrum but with a shuffled phase). Noise-related ICs are defined as those that are significantly correlated with the noise (*p* < 0.05 Bonferroni-corrected for multiple comparisons). Ideally, the ICA should produce noise ICs that are well correlated with the noise markers. Therefore, the performance of the ICA in noise-component identification is evaluated through the *Noise-identification effectiveness ratio*: defined as the ratio of the average variance explained by noise in noise-related ICs (R^2^ between the noise time series and the time course of the ICA-identified noise-related components) divided by the average variance explained by noise in non-noise components (R^2^ between the noise time course and the time course of the non-noise ICs). Higher ratio represents better performance.

Moreover, we also recorded the percentage of the components that have a significant correlation with the noise markers. Although a lower percentage of noise-related ICs does not necessarily indicate better performance, but if all other performance metrics are indistinguishable between sICA and tICA, then the method that achieves the performance by identifying and removing a lower percent of noise components is preferable, as it suggests more efficient noise identification that better preserves the degree of freedom. A lower percent represents better performance (Carone et al., 2017; De Blasi et al., 2020).

### Noise Removal

Noise-related and noise-non-related ICs are combined separately to reconstruct “noise” and “denoised” datasets. To generate denoised datasets, all columns in the mixing matrix that are identified as noise components are replaced with zeros, following which the data is reconstructed by multiplying the mixing matrix to the components matrix. Similarly, “noise” datasets are generated by reconstructing data after replacing the non-noise columns of the mixing matrix with zeros. Ideally, successful noise correction should result in a “cleaned” dataset that contains high brain functional connectivity information. Moreover, the denoising approach should not remove excessive variance from the original data.

#### Variance Retained and Removed

In this step, we used the output from the ICA step to generate “noise” and “denoised” datasets with regard to different noise types (as described earlier). That is, for each noise time series, we identified ICs that are significantly correlated with it, and combined them to produce the noise-specific signal contribution for that noise type. Conversely, the remaining ICs are combined to create the signal contribution that is not related to that particular noise type (“denoised data” with respect to that noise type). To evaluate the effectiveness of noise removal, variance in the fMRI data explained by each noise source is compared before and after ICA-based noise removal. To this end, voxel-wise R^2^ between each noise source and the original fMRI signal was calculated and the R^2^ values were averaged across the brain. The same process was followed for cleaned and noise datasets, whereby we defined the *Denoising effectiveness ratio* as R^2^(noise, designated noise ICs) / R^2^ (noise, denoised data). Successful noise removal would lead to a decrease in the R^2^ between the noise markers and denoised data while R^2^ between the noise markers and designated noise ICs should be high. Therefore. we expect to have a high R^2^ ratio for a successful noise removal.

Furthermore, a composite noise dataset is created by a weighted summation of all the ICs correlated with any of the noise markers, and a noise-free dataset is created by a weighted summation of the remaining components. The weights for each component are based on the estimated mixing matrix. Voxel-wise R^2^ between the original fMRI dataset and the composite noise and noise-free datasets is calculated and then averaged across the brain. This shows how much of the variance in the original fMRI signal is removed by correcting for all noise sources.

#### Effect on Functional Connectivity

To assess the effect of ICA denoising on functional connectivity, template-based rotation (TBR) (Schultz et al., 2014) is implemented on “cleaned” datasets to generate resting-state connectivity (rs-connectivity) maps for each individual using Yeo’s seven resting-state network (rs-network) templates (Yeo et al., 2011), namely the visual, somatomotor, dorsal attention, ventral attention, frontoparietal and default mode networks. Specifically, in TBR, functional volumes are described as a linear combination of network templates, and it is assumed that the network templates are meaningful segmentations of the rs-fMRI signal fluctuations. The first step of TBR is a spatial principal-component analysis of the fMRI data, resulting in mutually orthogonal principal components. These principal components are then mapped onto a network template using multi-regression. Thus, there is no requirement for the signals associated with individual network templates to be orthogonal. The same rs-fMRI image series could be used to map to multiple network templates, reflective of possible dependence amongst networks. The advantages for using TBR include that it provides more stable connectivity estimates as compared to traditional methods such as seed-based analyses, and that it offers a convenient means of incorporating the rs-fMRI network templates in our evaluation process.

Ideally, functional networks should be preserved in the “cleaned” images. As an example, group-average connectivity maps for the default mode network (DMN) are generated from TBR for the cleaned images. The following two measures are introduced to evaluate the presence of rs-networks in and “cleaned” data resulting from the ICA denoising stage:

##### Spatial Overlap With Known Functional Networks (Dice Coefficient)

Each network map generated using TBR is thresholded with a value that generates the maximum Dice coefficient with the functional-network template. The Dice coefficients are averaged across the six rs-networks (excluding limbic due to partial coverage and susceptibility noise). Ideally, concurrently high Dice coefficient from “cleaned” data demonstrates that the information about brain connectivity is preserved after noise correction (Table 1).

##### Intra-Extra Network Connectivity Difference (Δ*Z*)

For each network, the “cleaned” data is mapped to individual network templates using TBR, as described earlier. Subsequently, the average z-values (connectivity score) taken from outside each network is subtracted from the average within-network connectivity. Poor separability can result from poor data quality (Kong et al., 2020). Therefore, in a cleaned dataset we ideally expect to observe a greater difference between within-network and between-network connectivity.

### Statistical Test

Since the evaluation metrics are not always normally distributed, we used the paired-sample Wilcoxon signed-rank test to compare the metrics produced by the two methods (in addition to “no denoising”). To reduce false positives, p-values of less than 0.01 are considered to be significant (uncorrected).

## Results

### Simulations

**FIGURE 3:**
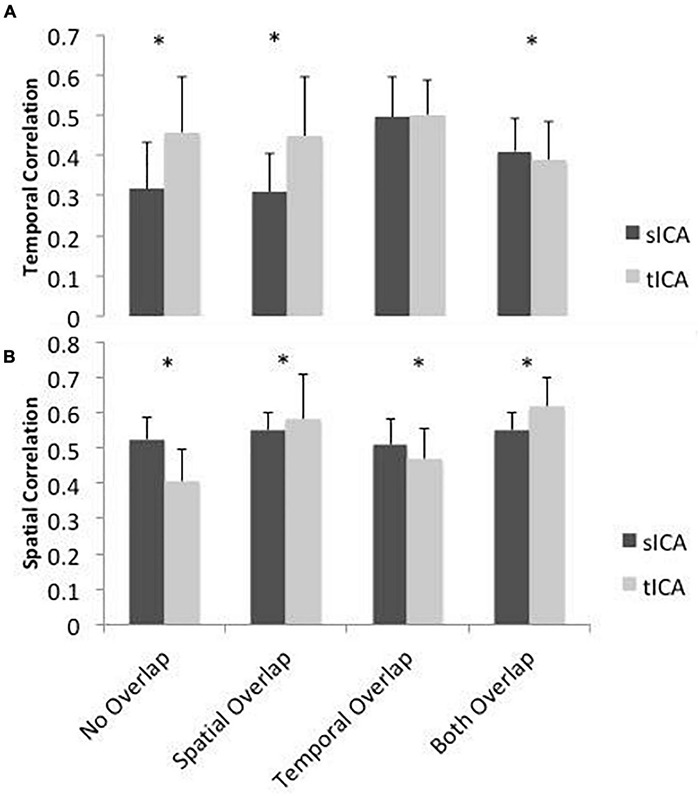
Simulated performance of sICA (black) and tICA (gray) in identifying signal components and spatial patterns. Performance is measured by calculating the correlation between the temporal **(A)** and spatial patterns **(B)** of the ground-truth and estimated components. Thus, a higher correlation indicates superior performance. When the components are spatially correlated, tICA performs better, whereas when the components are temporally inter-correlated, sICA results are more favorable. When components are both temporally and spatially correlated (“Both Overlap”), sICA results in higher performance in terms of temporal correlation with the ground truth, while tICA results in higher performance in terms of spatial correlation with the ground truth. Error bars show standard deviation across 100 simulations. Significant differences are indicated by asterisks (*p* < 0.05).

Results of the simulation are presented in Figure 3. For scenario 1 where the components are spatially and temporally uncorrelated, sICA outperforms tICA in identifying spatial patterns, whereas tICA can better identify the temporal patterns of the components. In scenario 2, whereby the components are spatially correlated, tICA displays better performance in identifying both spatial and temporal patterns of the components. In scenario 3, when the components are temporally correlated, sICA performs better in identifying the components’ spatial patterns. In Scenario 3, the performance of sICA and tICA in identifying the time-series of the components are comparable. In the scenario that the components are both spatially and temporally correlated, sICA can better identify components’ time series, while tICA can better identify components’ spatial patterns.

Overall, these simulations demonstrate that sICA performs better when the components are spatially uncorrelated and tICA performs better when the components signals have low temporal correlation, confirming our hypotheses.

### Experimental Data

As described earlier, each raw data set is divided into 30 ICs using either sICA or tICA. First, the performances of sICA and tICA in identifying noise components are compared using the two metrics explained in the first column of Table 1 (detailed in section “Materials and Methods”). The performance of spatial and temporal ICA in noise removal is then compared using the three metrics in the second column of Table 1 (detailed in section “Materials and Methods”). The distinction between the evaluations of noise identification and noise removal is that in the former case, we focus on the presence of noise contributions in the ICs of the original data identified as “noise-related”, whereas in the latter, we focus on the presence of noise contributions in the ICA-denoised data.

**FIGURE 4:**
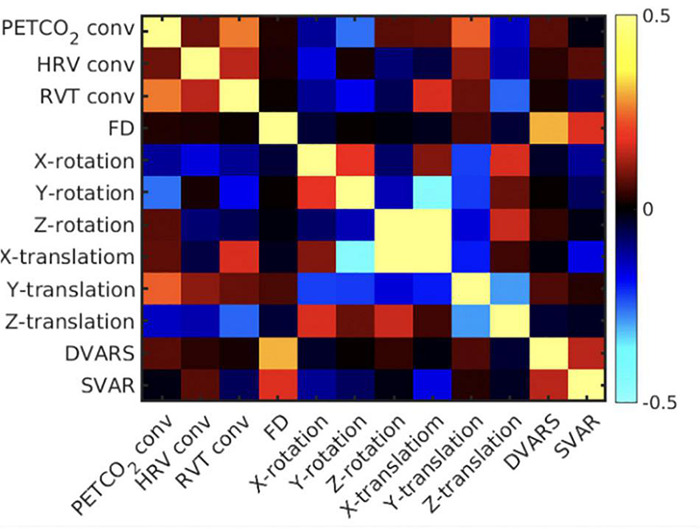
Mean correlation matrix across all pairs of noise regressors. The correlation matrix is the average across subjects. The colorbar represents correlation coefficients, and the diagonal are populated by 1’s.

To confirm the classification of the different noise sources, their temporal correlations are assessed for each subject, then averaged across subjects (Figure 4). PETCO_2_-conv, RVT-conv and HRV-conv have been convolved with their respective fMRI response functions, whereas the remaining regressors have not. Overall, local motion parameters (translation and rotation) are mutually correlated. For instance, there is high correlation between x- and z-translation. Moreover, y-rotation is negatively correlated with x-translation, and z-translation is negatively correlated with y-translation. On the other hand, global motion parameters such as FD and DVARS are moderately correlated, but they are not correlated with local motion parameters. Lastly, consistent with previous research (Chang and Glover, 2009), the convolved RVT and PETCO_2_ are also moderately correlated. Lastly, correlations between the physiological signals and local head motions are low to moderate (RVT-conv with X-rotation, PETCO_2_-conv with y-rotation). These results generally support the classification of local and global noise sources based on temporal correlation.

#### Noise Identification

**FIGURE 5:**
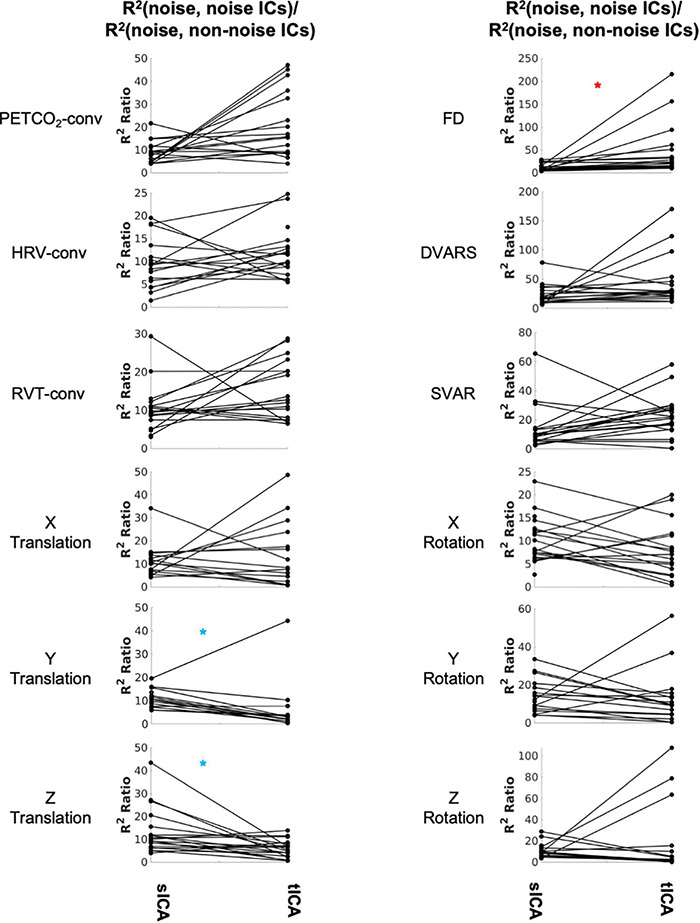
Comparing sICA and tICA in identifying noise components. Each line represents one subject. Each plot depicts the noise-identification effectiveness ratio, i.e., the ratio of R^2^ between noise markers and noise-correlated ICs over the average R^2^ between the noise markers and non-noise-correlated ICs. Significantly higher values for sICA are indicated by the blue asterisk, whereas significantly higher values for tICA are indicated by the red asterisk. The significance of the changes is indicated in boldface in Supplementary Table 1.

As shown in Figure 5 and Supplementary Table 1, the Wilcoxson signed-rank test revealed significantly fewer tICA components than sICA components that are correlated with noise sources (indicated by blue asterisks). However, the noise-related ICs identified by tICA are more distinct from the non-noise related ICs, as indicated by a higher noise-identification effectiveness ratio (Figure 5) for FD (red asterisk). In this respect (of the R^2^ ratio), sICA is only significantly advantageous in the cases of Y and Z translation (blue asterisks in the right columns).

#### Noise Removal

**FIGURE 6:**
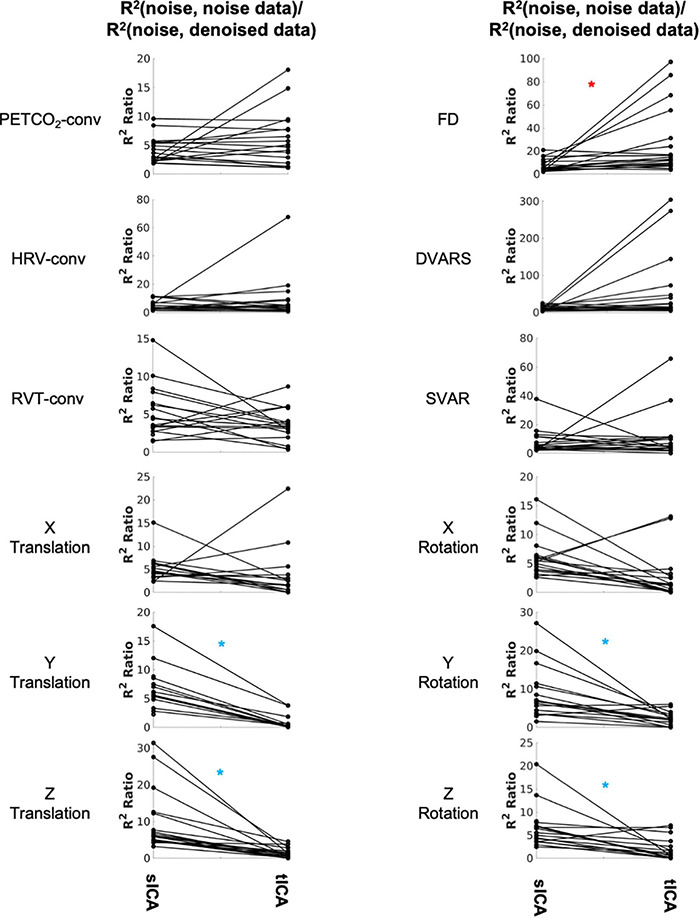
Comparing sICA and tICA in removing the effect of noise: the variance explained by noise type. Each line represents one subject. In each column, each plot shows the ratio of variance accounted for by each noise/artifact source in the noise-related signal identified by sICA and tICA over the corresponding variance in the ICA-denoised signal. A higher R^2^ ratio indicates more successful denoising to some extent. Significantly higher values for sICA are indicated by the blue asterisk, whereas significantly higher values for tICA are indicated by the red asterisks. The significance of the changes is indicated in boldface in Supplementary Table 2.

The noise components identified by both sICA and tICA have high shared variance (R^2^) with the noise sources, with a higher R^2^ ratio being indicative of higher denoising effectiveness (Table 1). By this metric, the performance of tICA for FD-related noise removal is superior to sICA (Figure 6, red asterisk), with the significance values summarized in Supplementary Table 2. For the motion-realignment (translation and rotation) parameters, sICA performance is more consistent and significantly superior, specifically for Y and Z translation and rotation (blue asterisks, details found in Supplementary Table 2). Similar findings pertain to the case of ICA producing 50 rather than 30 ICs (see Supplementary Material).

**FIGURE 7:**
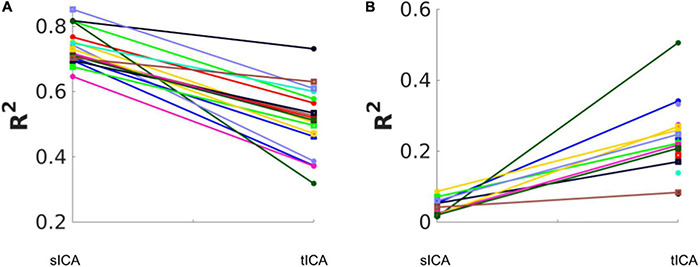
Total variance (R^2^) removed and retained by sICA and tICA. **(A)** The variance accounted for by the composite noise contributions generated from the noise-correlated ICs. **(B)** The variance accounted for by the composite non-noise contributions generated from the non-noise-correlated ICs. Each color represents one subject. The results show that sICA consistently removes more variance from the original signal than tICA.

The effect of removing all noise sources is shown in Figure 7. Subjects are coded with different colors and symbols. In both cases, all ICs that are significantly associated with any noise source are considered noise-related and removed in the denoising step. Overall, tICA preserved considerably more variance of the original data. In some cases, sICA removed up to 80% of the variance in the original data.

**FIGURE 8:**
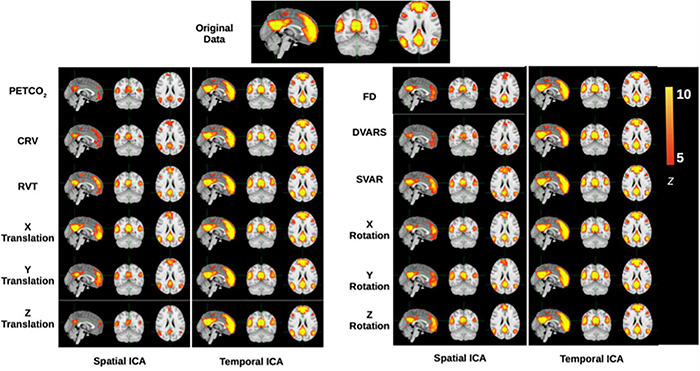
Functional-connectivity patterns associated with the denoised signals. Each row corresponds to the group-average TBR-based connectivity maps associated with signals that had specific noise correction applied to it. At the top is shown the DMN connectivity map obtained from the original data, and denoised data should maximally display DMN structure, which is generally stronger in tICA-cleaned signals.

To illustrate the influence on functional connectivity (FC), the DMN connectivity maps generated from corrected datasets are shown in Figure 8. The DMN generated from corrected data with tICA is more similar to the original DMN map, whereas the maps generated from corrected data with sICA have lower Z-values and, in some cases, missing nodes of the DMN network (e.g., dorsolateral-prefrontal node).

**FIGURE 9:**
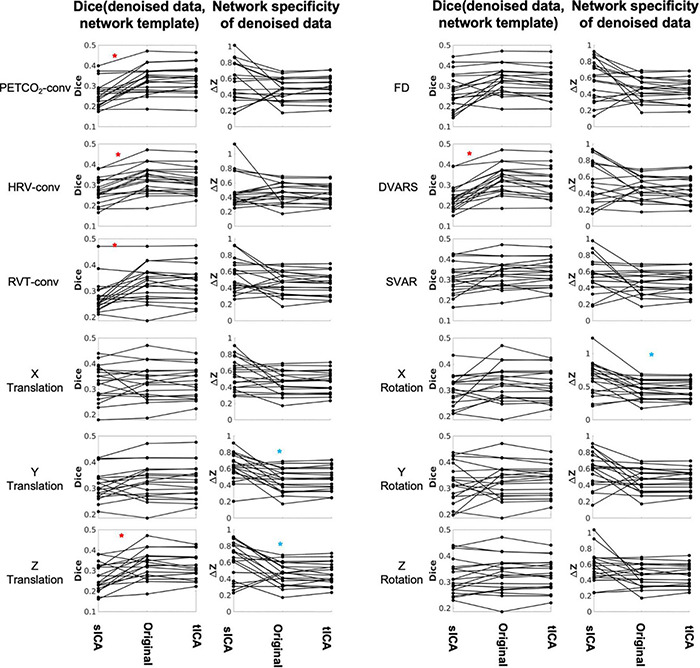
Comparing sICA and tICA in removing the effect of physiological signals: Spatial overlap of connectivity pattern with known functional networks (Column 1) and intra-extra network difference (Column 2). Each line represents one subject. Column 1 represents the average Dice coefficient between the rs-networks template and the resting-state network maps generated from denoised fMRI data. Column 2 represent functional specificity of each connectivity map as measured by calculating the average ΔZ between voxels within networks and those outside networks, and averaged over all networks. In each plot the ICA-denoising outcome is compared to the outcome from the original data (without ICA denoising). Significantly higher values for sICA are indicated by the blue asterisk, whereas significantly higher values for tICA are indicated by the red asterisks. The significance of the changes is indicated in boldface in Supplementary Tables 3, 4.

To quantify the FC comparisons, the Dice coefficient was used to gauge the spatial similarity between each IC and template functional networks (Figure 9 and Supplementary Table 3). As mentioned previously, six networks were considered, namely the visual, motor, default-mode, dorsal attention, ventral attention, and frontoparietal networks. In non-noise ICs (those uncorrelated with each of the individual noise markers), tICA-denoised TBR results are shown to have significantly higher Dice coefficients, specifically after removing PETCO_2_, HRV, or RVT effects.

Network structure in non-noise related rs-fMRI signal contributions were assessed as the intra-extra network connectivity difference (ΔZ) (shown in Figure 9 and Supplementary Table 4). Compared to tICA, sICA denoising resulted in more variable changes in the network specificity. sICA exhibits greater inter-subject variability, as indicated by the spread of the Dice and network specificity metrics. The Dice coefficient (network-template overlap) is significantly higher for data after tICA-based removal of the effects of PETCO2, HRV, RVT, Z translation and DVARS (red asterisks), whereas network specificity (ΔZ) is significantly higher after sICA-denoising X rotation as well as Y and Z rotation (blue asterisks). Similar findings pertain to the case of ICA producing 50 rather than 30 ICs (see Supplementary Material), demonstrating the generalizability of these findings.

The performance of tICA and sICA in identifying and removing noise sources are summarized in Table 2. Our results agree with the simulation-based predictions. Specifically, tICA performs better in identifying and removing global components with high spatial correlations (FD, DVARS, PETCO2, RVT, and HRV), whereas sICA is more successful in identifying and removing components that are temporally correlated, but are spatially localized (local head motion parameters).

**TABLE 2:** Summary of performance of sICA vs. tICA for various noise markers.

Performance category		sICA outperforms tICA	tICA outperforms sICA	sICA and tICA equivalent
**Noise identification**	**By R^2^ ratio (**Figure 5)	y-translation z-translation	FD	PETCO2 RVT HRV DVARS SVAR x-translation x-rotation y-rotation z-rotation
**Noise removal**	**By R^2^ ratio (**Figure 6)	y-translation z-translation y-rotation z-rotation	FD	PETCO2 RVT HRV DVARS SVAR x-translation x-rotation
	**By connectivity accuracy (**Figure 9)		PETCO2 RVT HRV Z-translation DVARS	FD SVAR x-translation y-translation x-rotation y-rotation z-rotation
	**By connectivity specificity (**Figure 9)	y-translation z-translation x-rotation		PETCO2 RVT HRV DVARS SVAR FD x-translation y-rotation z-rotation

## Discussion

Independent component analysisis a main-stream method of noise removal in rs-fMRI (Salimi-Khorshidi et al., 2014; Pruim et al., 2015). As ICA denoising can be purely data-driven, it circumvents the lack of physiological and motion recordings in many large-scale studies. However, to date, most ICA-related rs-fMRI studies have opted for sICA (Calhoun et al., 2001; Beckmann et al., 2005; Smitha et al., 2017), leaving tICA underexplored. We are cognizant of the rising use of accelerated rs-fMRI acquisitions (Lee et al., 2013; Preibisch et al., 2015; Demetriou et al., 2018), which is making tICA in rs-fMRI an increasing possibility. The effectiveness of tICA in identifying and removing the more global RVT effects in a group-wise tICA implementation has been shown (Glasser et al., 2018). In this study, we compare the performances of sICA and tICA for denoising rapidly sampled rs-fMRI data. Importantly, as we also have physiological and motion time series at our disposal, we are able to evaluate both types of ICA using these time series as reference rather than rely solely on more subjective evaluation. That is, noise-related ICs were identified based on significant correlation with noise parameters rather than based on spatial pattern or frequency distribution (Bhaganagarapu et al., 2013; Sochat et al., 2014). Furthermore, we use the available noise parameters to segregate the data into substrates driven by different noise types, namely physiological and motion.

In this study, although the spatial resolution (4 mm × x4 mm × 5 mm) is lower than in studies such as the Human Connectome Project (HCP) (Glasser et al., 2013), such spatial resolutions are not uncommon amongst legacy data that are still being actively analyzed (Glover, 2011). Moreover, we traded spatial resolution to achieve a much higher sampling rate (TR = 0.38 s), the intention being to help us avoid the brunt of aliasing high-frequency physiological noise in the f < 0.1 Hz band. This is an important advantage of our dataset over the HCP project, where cardiac signal is not critically sampled and therefore is aliased into low frequency bands. It bears mentioning that we used a simulation-assisted approach to support our conclusions, an approach we have consistently embraced in our work (Chu et al., 2018; Yuen et al., 2019). Thus, the availability of comprehensive physiological recordings in our study enabled hypothesis testing, by clarifying the temporal relationships amongst the many noise markers (Figure 4). In this study we assume that noise consists of all known signals in the frequency band <0.1 Hz that have non-neural origins, including low-frequency physiological variability and head motion. Figure 4 confirms three points: (1) the local affine motion parameters (rotations and translations) are mutually correlated temporally; (2) the global motion parameters such as FD and DVARS are not temporally correlated with these local motion parameters; (3) physiological processes introduce temporally distinct effects from both of these categories of noise markers. It is thus clear that neither sICA nor tICA is ideal for addressing all of these types of noise, and a deeper understanding of the performances of sICA and tICA is key to understanding the natures of the diverse noise sources in the low-frequency band.

### Comparison of Denoising Methods: Denoised Resting-State fMRI Signal Content

Temporal ICA and sICA denoising behave very differently, although it is customary to precede tICA with sICA-based dimensionality reduction, as stated earlier (Smith et al., 2012; Glasser et al., 2018). The first main finding of this study is that tICA is likely to identify fewer noise ICs than does sICA for our fMRI data. At the same time, the noise-related ICs identified by sICA and tICA are similarly associated with the noise time courses with respect to the noise-identification effectiveness ratio, tICA achieves this with a lower loss in degrees of freedom and potentially preserves more neuronally meaningful signal contributions. More specifically, as shown in Figure 6, while tICA performs significantly better for isolating the effect of FD, sICA performs better for isolating the influences of Y and Z affine motion realignment parameters. Furthermore, once all noise-associated ICs are removed, we found sICA removes much more variance from the original signal than tICA, as demonstrated in Figure 7; this is the second main finding of this study.

Framewise displacement has a more global signature, as it is calculated from the whole-brain average signal, and acts like the summation of all affine motion parameters. On the other hand, the effect of the affine head motions can contain aspects that are localized at the edge of the brain with limited spatial overlap with brain networks and other signal sources. Thus, following the scenarios addressed in the simulations, tICA, as expected, performs better for isolating physiological noise (which share spatial distributions with brain networks), whereas sICA, as expected, performs better for noise types that have lower spatial overlap with brain networks. Nonetheless, we still need to assess whether these differences translate into equivalent performance differences in functional connectivity mapping. Lastly, the inter-subject variability of the performances of tICA and sICA are largely comparable, both exhibiting high levels of variability. This is also unsurprising, as the spatial signature of physiological noise varies greatly across subjects (Bianciardi et al., 2009; Chang and Glover, 2009; Falahpour et al., 2013).

### Comparison of Denoising Methods: Resting-State fMRI Connectivity

We found that sICA-denoised data is associated with lower network structure than tICA-denoised data, as shown in Figure 8 for the case of the DMN. When summarized across multiple brain networks, as the Dice coefficient reflects the degree of overlap between the network templates and the TBR maps of functional connectivity in the denoise data, a higher Dice coefficient in the denoise part of the signal is more ideal. Based on this metric, tICA denoising of physiological (PETCO_2_, HRV, and RVT), DVARS and Z translation effects resulted in higher network integrity than sICA denoising (Figure 9). Similar findings pertain to the case of ICA producing 50 rather than 30 ICs (see Supplementary Material), demonstrating the generalizability of these findings. This is consistent with the finding that sICA denoising removes more variance from the data compared to tICA, part of which contributes to legitimate functional connectivity. These findings can be justified particularly as physiological (PETCO_2_, HRV, and RVT) and DVARS share regions of influence (Tong et al., 2015; Bright et al., 2020). Since sICA prefers spatial independence amongst ICs, it is less able to disentangle the effect of these noises from the underlying connectivity signals and therefore partially removes information about brain connectivity. On the other hand, sICA-denoisation data produces higher network specificity after removing X rotation and Y/Z translation effects, as these noise sources are likely to produce more spatially localized effects (Pruim et al., 2015).

The findings of this study are summarized in Table 2 for an easier overview. Consistent with our hypothesis and the simulation results, sICA performs better in identifying and removing spatially uncorrelated components such as rotations and translations. The superiority of the sICA is more pronounced in removing the components that exhibit stronger temporal rather than spatial correlations (e.g., y-rotation, y-translation, z-translation in Figures 5, 6). On the other hand, noise markers that have strong spatial overlap with other noise markers or brain networks can be removed more efficiently by tICA; these include PETCO_2_, HRV, RVT, FD, and DVARS. Lastly, tICA results are associated with lower inter-subject variability in spatial specificity (ΔZ) than sICA results, as seen in Figure 9. It should be noted that the metrics used to compare the performance of sICA and tICA noise removal show complementary aspects of each method’s performance, therefore the evaluation should consider the various quality metrics simultaneously.

Taken together, our findings suggest that there is real merit in considering an ICA-hybrid approach in which physiological and motion-related noise are each identified using tICA and sICA, respectively. This study is a first step toward recognizing the importance of such a hybrid approach. The objective of this study was to show the differences of tICA and sICA in identifying and removing individual noise contributions. Future studies are required to move towards developing and optimizing a hybrid approach by answering such questions as how to address interdependencies amongst the noises and the sequence of tICA vs. sICA for noise removal.

### Limitations

In this work we made a key assumption that noise in rs-fMRI should exhibit different statistical distributions from the signal. In reality, statistical distributions of physiological processes are not entirely distinct from those observed within functional networks. However, the inter-subject variability in these effects is very high (Chang and Glover, 2009; Golestani et al., 2015), creating large uncertainties as to the overlap with functional networks on a per-subject basis. The standard deviation of respiratory depth (RV) instead of RVT may be more robust against measurement artifacts (Glasser et al., 2018; Power et al., 2019a). As a result, the frequency occupancy of these signals can be leveraged to separate them from neuronally driven signals to some degree (Yuen et al., 2019). Nonetheless, this work presumes the currently dominant view that physiological processes are part of the “noise”. Further investigations are underway to verify that claim.

Furthermore, fMRI data typically has a much higher spatial than the temporal dimension. This leads to instability when applying tICA. To overcome this problem, it is inevitable to reduce the spatial dimensionality of the data. This is typically done using principal component analysis (PCA) (Calhoun et al., 2001; Boubela et al., 2013) or an initial spatial ICA (Smith et al., 2012; Glasser et al., 2018). In this study we performed an initial sICA-based data reduction, which is a common step in the tICA approach in fMRI (Smith et al., 2012; Glasser et al., 2018), to reduce the spatial dimension of the data to 100, which is a typical spatial space of the fMRI data (Craddock et al., 2012; Glasser et al., 2016a). Therefore, tICA as it is typically reported is in fact a mixed method, which uses both sICA and tICA. We did not test other dimension reduction methods or using sICA with a different dimension. Further studies are required to investigate whether different dimension reduction approaches would alter the findings.

Lastly, the spatial resolution of the current data is lower than used in the state-of-the-art studies (Glasser et al., 2016b; Miller et al., 2016). However, it is still representative of the numerous sets of legacy data (Teipel et al., 2017; Kraus et al., 2020), with an important advantage of critically sampling cardiac and respiratory noise peaks, which is not possible with online public datasets, like the Human Connectome Project. This latter allows us a unique advantage to decipher the effects of low-frequency artifacts, although this results in the use of a lower than usual flip-angle (40 degrees). While such a low flip angle minimizes the contribution of physiological noise (Gonzalez-Castillo et al., 2011), given the loss in image SNR brought about by the short TR, the overall temporal SNR is no higher than at a higher flip angle (70–90°). The TR-driven temporal-SNR can influence the performance of ICA, and should be investigated as the next step.

## Data Availability Statement

The raw data supporting the conclusions of this article will be made available by the authors, without undue reservation.

## Ethics Statement

The studies involving human participants were reviewed and approved by Research Ethics Board of Baycrest. The patients/participants provided their written informed consent to participate in this study.

## Author Contributions

AG: conceptualization, data acquisition, data analysis, and manuscript preparation. JC: data acquisition, supervision of data analysis, and manuscript preparation. Both authors contributed to the article and approved the submitted version.

## Conflict of Interest

The authors declare that the research was conducted in the absence of any commercial or financial relationships that could be construed as a potential conflict of interest.

## Publisher’s Note

All claims expressed in this article are solely those of the authors and do not necessarily represent those of their affiliated organizations, or those of the publisher, the editors and the reviewers. Any product that may be evaluated in this article, or claim that may be made by its manufacturer, is not guaranteed or endorsed by the publisher.
